# KRT17 Accelerates Cell Proliferative and Invasive Potential of Laryngeal Squamous Cell Carcinoma (LSCC) through Regulating AKT/mTOR and Wnt/*β*-Catenin Pathways

**DOI:** 10.1155/2022/6176043

**Published:** 2022-10-05

**Authors:** JianQiu Wang, Longjiang Lan, Bingliang Ma, Gang Ren, ChengYi Yin

**Affiliations:** Department of Otolaryngology, The First Affiliated Hospital, Huzhou University, The First People's Hospital of Huzhou, Huzhou 313000, Zhejiang, China

## Abstract

**Background:**

Laryngeal squamous cell carcinoma (LSCC) is a prevalent malignant tumor of the head and neck with a dismal prognosis. Keratin17 (KRT17) has been proven to serve as an oncogene in various cancers, but it has never been explored in LSCC. We proposed to assess the impact and possible mechanisms of KRT17 in the development of LSCC.

**Methods:**

Quantitative reverse transcription-PCR (qRT-PCR) was utilized to examine the mRNA levels. The Kaplan–Meier method was used to calculate the relationship between KRT17 expression and survival curves in LSCC patients. Cell counting kit-8 (CCK-8), colony formation, and flow cytometry assays were utilized to estimate LSCC cell proliferation. The migration and invasion abilities of LSCC cells were ascertained by wound-healing and transwell assays. Immunohistochemical and western blot assays were utilized to appraise protein levels. The xenograft tumor model was used to determine the effect of KRT17 on tumor growth.

**Results:**

In the present study, KRT17 was extremely high in LSCC tissues and cells and correlated with a poor prognosis. Inhibition of KRT17 weakens cell proliferative, migratory, and invasive abilities in LSCC and contributes to cell cycle arrest. Besides, we approved that knockdown of KRT17 extraordinarily restrained the xenograft tumor growth in vivo. We preliminarily investigated the role of KRT17 on the AKT/mTOR and Wnt/*β*-catenin signaling axes and found that these signaling pathways were largely blocked by KRT17 deletion.

**Conclusion:**

Collectively, we uncovered that exhaustion of KRT17 suppresses LSCC progression through coordinating AKT/mTOR and Wnt/*β*-catenin signaling axes, illustrating KRT17 as a promising biomarker for making strides in LSCC treatment.

## 1. Introduction

Laryngeal squamous cell carcinoma (LSCC) is the second largest sort of head and neck malignancy, with extending recurrence and mortality [1–3]. In spite of the ceaseless enhancement of treatment and demonstrative strategies, the overall 5-year survival rate for LSCC patients remains unsatisfactory, especially for those with advanced or metastatic disease [4, 5]. Subsequently, there is a pressing need to address the essential molecular regulatory pipelines of LSCC pathogenesis.

Keratin, one of the intermediate filament of the protein family, has a molecular weight of about 40–70 kDa and forms the cytoskeleton [6, 7]. Keratin can be separated into sort I keratin and sort II keratin, of which KRT17 is commonly found in epithelial cells and belongs to sort I keratin [8, 9]. KRT17 is not present in the epidermis of normal skin but can be initiated under stressful conditions such as skin scratching [10]. Studies have confirmed that KRT17 is a typical marker of hyperproliferation in psoriatic skin, and extracts of *Curcuma amada*, *Humulus lupulus,* and *Hypericum perforatum* can inhibit the expression of KRT17 in psoriatic skin [11]. KRT17, as a multifunctional promoter and oncogene, has appeared to play an imperative role in advancing the proliferation, metastasis, and consequent deadly results of malignant tumors [12–16]. In the meantime, other confirmations have also appeared that KRT17 expression was expanded in tumor tissues but not in nontumor regions [17]. In addition, the high expression level of KRT17 has been shown to be closely relevant to the advancement of epithelial-mesenchymal transformation (EMT), proposing another crucial portion in accelerating cancer cell survival and metastasis [18]. A later study utilized RNA sequencing for gene expression profiling in human normal mucosal and LSCC tissues and recognized 50 genes with the highest upregulation in LSCC, of which KRT17 was described as one [19]. In any case, it is not explicit whether KRT17 can play a part in LSCC and its mechanism.

In this study, we uncovered that KRT17 is elevated in LSCC tissues and is possibly associated with poor outcomes. KRT17 accelerates LSCC proliferation and invasion by means of enacting AKT/mTOR and Wnt/*β*-catenin signaling axes. Our revelations propose that KRT17 may be an imperative marker of LSCC progression and unfavorable survival.

## 2. Materials and Methods

### 2.1. Patient Samples

A total of 42 human LSCC and correspondent nontumor normal samples (5 cm distant from the tumor boundary) were obtained from the LSCC patients who experienced surgical resection in the First Affiliated Hospital, Huzhou University, The First People's Hospital of Huzhou hospital. All participants did not undergo preoperative radiotherapy or chemotherapy and signed an informed consent form. This study was endorsed by the Institutional Ethics Committee of the First Affiliated Hospital, Huzhou University, The First People's Hospital of Huzhou Hospital, and was undertaken in compliance with the Declaration of Helsinki.

### 2.2. Quantitative Reverse Transcription-PCR (qRT-PCR)

The cells and tissues were lysed utilizing Trizol reagent (Sangon Biotech (Shanghai) Co., Ltd., China). The PCR examination was accomplished utilizing PrimeScript RT reagent kit (Takara, Dalian, China) and SYBR Premix ExTaq II (TaKaRa). The primer sequences are included in Table 1.

A PPI network was constructed using the STRING (https://string-db.org) database based on key KRT17 coregulated genes in order to assess potential interactions between proteins of interest [20]. Interactions with a medium confidence score ≥0.4 were considered as significant.

### 2.3. Western Blot

A RIPA lysate (Beyotime, China) was utilized to extricate total proteins, which were quantified by a BCA kit (Solarbio, China), followed by subjecting them to an SDS-PAGE gel and transferring them to PVDF membranes (GE Healthcare Life, USA). Afterwards, blocking blots were performed with 5% fat-free milk, and the process was followed by incubation with the primary antibodies against KRT17 (AF5480, Affinity, USA), E-cadherin (AF0131, Affinity), N-cadherin (AF4039, Affinity), *β*-catenin (AF6266, Affinity), AKT (AF6261, Affinity), p-AKT (AF0016, Affinity), mTOR (AF6308, Affinity), p-mTOR (AF3308, Affinity c-Myc (AF0358, Affinity), cyclin D1 (AF0931, Affinity), cyclin E (AF0144, Affinity), CDK2 (AF6237, Affinity), CDK4 (DF6102, Affinity), Snail (AF6032, Affinity), MMP7 (AF0218, Affinity), and GAPDH (AF7021, Affinity). Then, the membranes were encoded with a secondary antibody conjugated to HRP and finally subjected to measurement with an ECL kit (Pierce, Waltham, MA, USA).

### 2.4. Cell Culture Conditions and Transfection Procedure

Human LSCC cells (TU686, TU177, AMC-HN-8, and TU212) and bronchial epithelioid cells (16HBE) were obtained from iCell Bioscience Inc. (Shanghai, China) and cultured under the appropriate media and conditions as required by the instructions. Following the operational requirements of Lipofectamine 2000 (Invitrogen, Carlsbad, CA, USA), KRT17 shRNAs, and an appropriate negative control (GenePharma, Shanghai, China) were transfected into AMC-HN-8 and TU177 cells.

### 2.5. Cell Counting Kit-8 (CCK-8) Assay

5000 AMC-HN-8 and TU177 cells were inoculated in each well of a 96-well plate, followed by the addition of 10 *μ*l CCK-8 (Beyotime) at the time of 24, 48, 72, and 96 h of incubation and then incubated for 4 h and detection of absorbance values at 450 nm using an enzyme reader (Molecular Devices, USA).

### 2.6. Colony Formation Assay

AMC-HN-8 and TU177 cells were inoculated into a 6-well plate and cultured for 2 weeks until the cells shaped into large clones. The complete medium was supplanted every 3 days. According to the requirements of crystal violet staining, clones were dyed, counted, and photographed.

### 2.7. Cell Cycle Analysis

AMC-HN-8 and TU177 cells were cultivated for 48 h, harvested by centrifugation, and fixed with 70% ethanol at 4°C for 12 h. DNA staining solution (propidium iodide and RNase A, BD Biosciences, USA) and 5 *μ*L osmotic solution were included under dark conditions, and cell cycle distribution detection was conducted using a flow cytometer (BD Biosciences).

### 2.8. Cellular Wound-Healing Assay

When 6-well plate cell growth reached 90% confluence, the scratches were delivered with a 200 ul pipette tip and cultured without fetal bovine serum, and the gap size was measured 24 h later.

### 2.9. Transwell Assay

The abilities of cells to migrate or invade were determined in transwell chambers (Corning, USA) without or pre-surfaced Matrigel. The AMC-HN-8 and TU177 cells (1 × 10^5^ cells/well) were included in the top chambers without serum medium, whereas the complete medium was included in the bottom chambers. After 24 h of incubation, cells moving to the bottom chambers were fixated with 4% paraformaldehyde, subsequently dyed with crystal violet, counted, and photographed.

### 2.10. Tumor Xenograft Model

This study involved animal experiments that were endorsed by the institutional guidelines of the Committee of the First Affiliated Hospital, Huzhou University, The First People's Hospital of Huzhou Hospital, and were undertaken in compliance with the Declaration of Helsinki. 2 × 10^6^ LSCC cells transfected with sh-KRT17 or sh-NC were separately injected into the right flank of each female nude mouse with 6 mice (4–5 weeks old) randomized in each group. Tumor volume was monitored at 3-day intervals utilizing a vernier caliper. Thirty-one days after injection, tumors were resected and weighed. Then, tumor tissues were fixed in formalin, embedded in paraffin, and cut into 4-*μ*m-thick slices. The sections were incubated with the primary anti-Ki-67 (AF0198, Affinity) and anti-KRT17 (AF0190, Affnity) antibodies at 4°C for 12 h after dewaxing and antigen repair. In this way, HRP-conjugated secondary antibody was added, and the signals were generated with diaminobenzidine and photographed with microscopy.

### 2.11. Statistical Analysis

Data are stated in terms of mean ± SD from at least three individual replicate trials. Statistics were obtained with SPSS 16.0 software, and graphical plots were generated with GraphPad Prism 8.0 software. Differences between two or among more groups were performed via Student's *t*-test or one-way analysis of variance (ANOVA). LSCC patients were assigned to either low or high KRT17 expression groups based on the median value of KRT17 expressed in LSCC tissues, and their overall survival curves were ascertained following the Kaplan–Meier method. *P* < 0.05 was affirmed as representing statistical significance.

## 3. Results

### 3.1. KRT17 Expression Is Elevated in LSCC Tissues and Cells and Is Linked to Poor Prognosis

**Figure 1:**
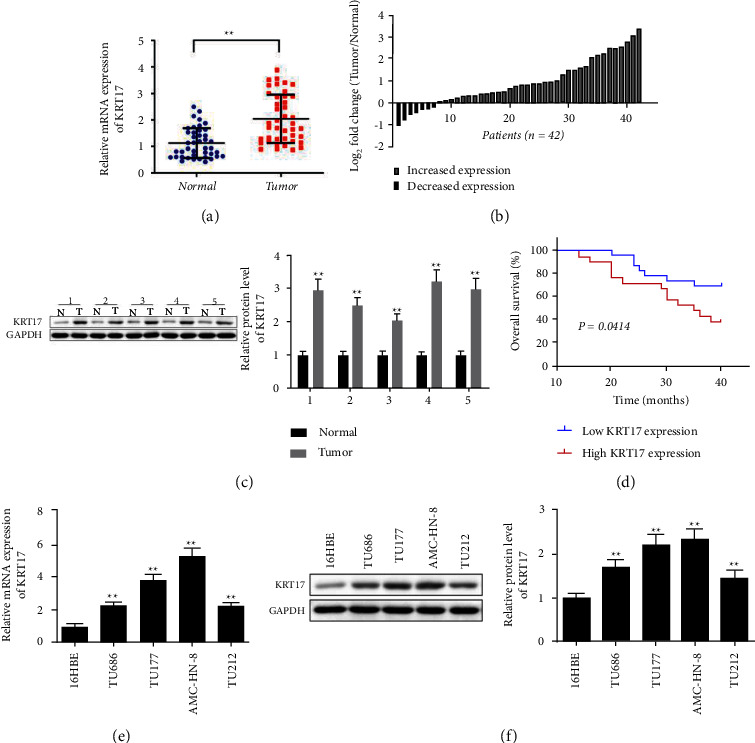
KRT17 expression is elevated in LSCC tissues and cells and is linked to poor prognosis. (a, b) QRT-PCR detected KRT17 mRNA expression in LSCC and nontumor tissues (*n* = 42). (c) Western blot detected KRT17 protein level in LSCC and nontumor tissues (*n* = 5). (d) Overall survival of LSCC patients with high or low level of KRT17. (e) qRT-PCR assessed KRT17 mRNA in AMC-HN-8, TU686, TU177, TU212, and 16HBE cells. (f) Western blot detected KRT17 protein level in AMC-HN-8, TU686, TU177, TU212, and 16HBE cells. ^*∗*^A significant difference compared with the normal group or 16HBE group. ^*∗∗*^*P* < 0.01. Abbreviations: N, nontumor tissues; T, LSCC tissues.

Previous studies have appeared that increased KRT17 expression in LSCC tissues [19]. Consistently, KRT17 mRNA expression in LSCC tissues was essentially enhanced by contrast to nontumor tissues (Figure 1(a)). In comparison to nontumor tissues, KRT17 mRNA expression was relatively low in 7 LSSC tissues, while KRT17 mRNA expression was relatively high in 35 LSSC tissues (Figure 1(b)). Western blot also confirmed the reliable result that KRT17 is elevated in LSCC tissues (Figure 1(c)).

Analysis of the correlation between KRT17 and clinic-pathological features of LSCC patients (Table 2) uncovered that KRT17 expression was remarkably correlated with differentiation (*P* < 0.001), T classification (*P* < 0.01), lymph node metastasis (*P* < 0.05), and clinical stage (*P* < 0.05). In any case, gender and primary position were not associated with KRT17 expression. To assist in assessing the clinical importance of KRT17 expression in LSCC, survival curves were utilized to compare the difference in overall survival between low and high KRT17 expression groups (*P*=0.414). The results uncovered that LSCC patients with high KRT17 expression had significantly lower overall survival than those with low KRT17 expression (Figure 1(d)). Furthermore, the KRT17 mRNA and protein levels were significantly overexpressed in AMC-HN-8, TU686, TU177, and TU212 cells with respect to the noncancer 16HBE cells (Figures 1–(e)1(f)). Since the expression of KRT17 was higher in AMC-HN-8 and TU177 cells, they were chosen for subsequent knockdown experiments.

### 3.2. Depletion of KRT17 Represses the Growth of LSCC Cells

**Figure 2:**
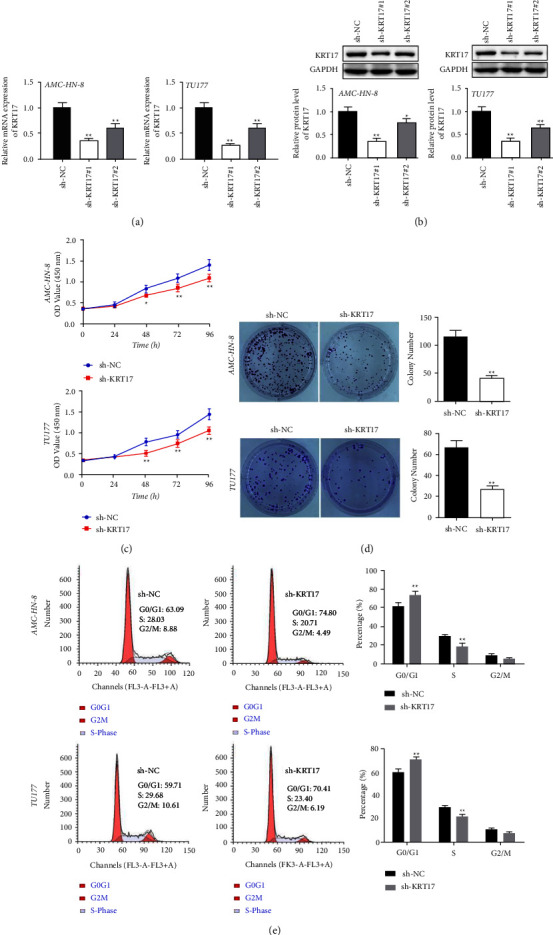
Depletion of KRT17 represses the growth of LSCC cells. (a) KRT17 was silenced by shRNAs determined by RT-qPCR. (b) The KRT17 protein level was estimated by western blot. (c) Cell viabilities were ascertained using CCK-8 assay. (d) The number of clones formed by AMC-HN-8 and TU177 cells was checked by a 2-week colony formation assay. (e) The cell cycle distribution ratios were determined analyzed using flow cytometry. ^*∗*^A significant difference compared with the sh-NC group. ^*∗*^*P* < 0.05, ^*∗∗*^*P* < 0.01, and ^*∗∗∗*^*P* < 0.001.

The findings of qRT-PCR and western blot appeared to show that sh-KRT17#1 and sh-KRT17#2 effectively diminished the expression of KRT17 both in AMC-HN-8 and TU177 cells (Figures 2(a)-2(b)), and sh-KRT17#1 was selected for and sh-KRT17#1 was chosen for subsequent trials due to its high knockdown efficiency. The CCK-8 and colony assays uncovered that inhibition of KRT17 in AMC-HN-8 and TU177 cells exhibited decreased proliferative capacities (Figures 2(c)–2(d)). Moreover, we performed a flow cytometry assay to determine the impact of sh-KRT17 on the LSCC cell cycle. The results implied that in AMC-HN-8 and TU177 cells, the cell cycle was stalled in the G1 phase after KRT17 knockdown (Figure 2(e)).

### 3.3. Depletion of KRT17 Hinders LSCC Cell Xenograft Tumor Growth *In Vivo*

**Figure 3:**
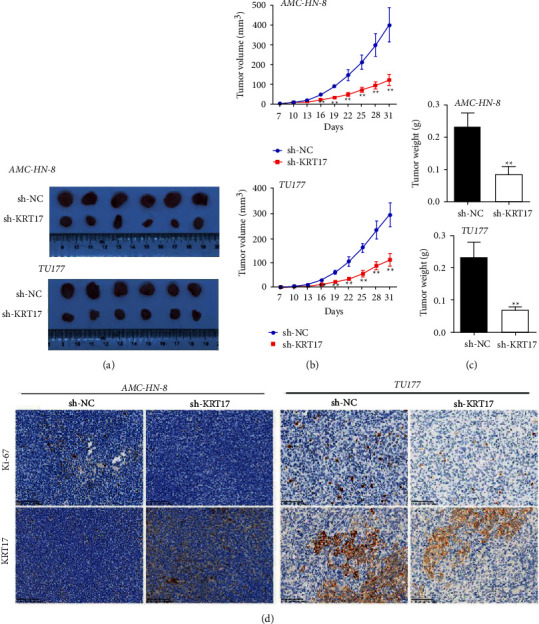
Depletion of KRT17 hinders *in vivo* LSCC cell xenograft tumor growth. (a) Images of tumor xenografts of mice injected with AMC-HN-8 and TU177 cells. (b) Tumor volume curves at 3-day intervals. (c) Tumor weight of mice tumor xenografts after 31 days after inoculation. (d) Representative immunohistochemical images of Ki-67 and KRT17 in tumor xenografts (scale bar = 100 *μ*m). ^*∗*^A significant difference compared with the sh-NC group. ^*∗*^*P* < 0.05 and ^*∗∗*^*P* < 0.01.

The results of nude mouse xenograft models transfected with AMC-HN-8 and TU177 cells exhibited dramatically lower tumor volume and weight in the sh-KRT17 group than in the sh-NC group (Figures 3(a)–3(c)). At the same time, the immunohistochemical assay proposed that the levels of Ki-67 and KRT17 were markedly down-regulated in the sh-KRT17 group in contrast to the sh-NC group (Figure 3(d)). Collectively, these findings uncovered that knockdown of KRT17 hinders tumorigenesis in an in vivo LSCC cell xenograft model.

### 3.4. Depletion of KRT17 Represses Migratory and Invasive Abilities of LSCC Cells

**Figure 4:**
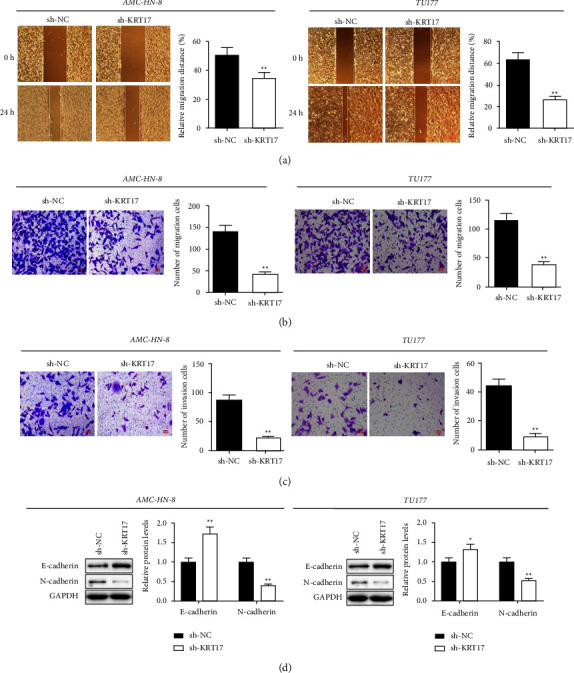
Depletion of KRT17 represses migratory and invasive abilities of LSCC cells. (a) Cell migratory abilities were assessed via the wound-healing assay (scale bar = 200 *μ*m). (b, c) Migratory and invasive abilities was examined using Transwell assay (scale bar = 50 *μ*m). (d) Protein levels were estimated by Western blot. ^*∗*^A significant difference compared with the sh-NC group. ^*∗∗*^*P* < 0.01.

Wound healing data revealed that AMC-HN-8 and TU177 cells depleted of KRT17 had significantly reduced scratch healing abilities compared to sh-NC cells, indicating slower cell motility. The metastatic capacities of AMC-HN-8 and TU177 cells were further detected by a transwell assay, which showed a significant reduction in the number of migrating and invading cells after the knockdown of KRT17. Meanwhile, downregulation of KRT17 essentially expanded the expression of E-cadherin but hindered N-cadherin compared with the sh-NC group (Figure 4(d)).

### 3.5. KRT17 Regulates LSCC Progression by Affecting AKT/mTOR and Wnt/*β*-Catenin Axes

**Figure 5:**
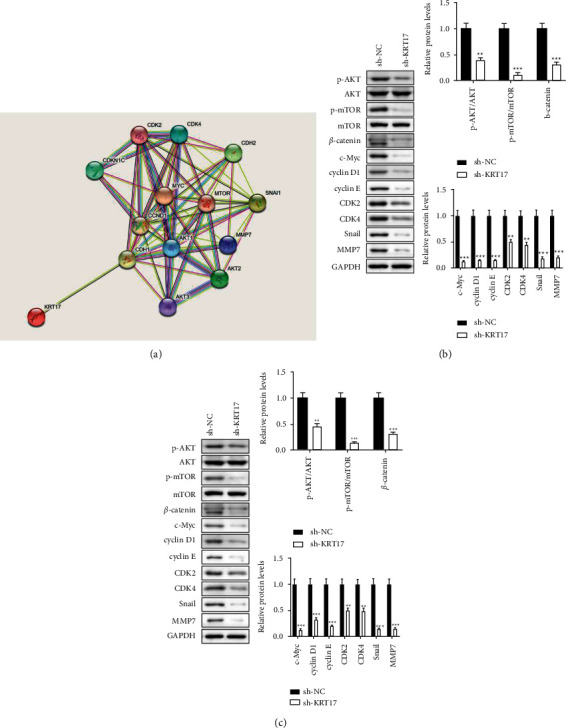
KRT17 regulates LSCC progression by affecting AKT/mTOR and Wnt/*β*-catenin axes (a). The protein-protein interaction (PPI) network of the KRT17 from STRING online database (https://string-db.org) was constructed (b). The protein levels in AMC-HN-8 cells were evaluated using western blot (c). The protein levels in TU177 cells were estimated using western blot. ^*∗*^A significant difference compared with the sh-NC group. ^*∗∗*^*P* < 0.01 and ^*∗∗∗*^*P* < 0.001.

Next, we explored the mechanism by which KRT17 mediates LSCC progression. The previous pieces of evidence have unraveled the accelerating impacts of KRT17 on AKT/mTOR and Wnt/*β*-catenin axes [21–24]. To further explore the effects of KRT17 in the AKT/mTOR and Wnt/*β*-catenin signaling pathway, we used the STRING database (https://www.string-db.org/) to identify KRT17-related genes. As shown in Figure 5(a), we generated a PPI network with a total of 14 nodes containing KRT17 and AKT/mTOR and Wnt/*β*-catenin signaling-related proteins. Subsequently, we verified KRT17-mediated changes in the levels of proteins related to the above pathways in AMC-HN-8 and TU177 cells by western blot assay. The results recommended that exhaustion of KRT17 restrained the critical proteins of AKT/mTOR and Wnt/*β*-catenin pathways (Figures 5(b)-5(c)). Depletion of KRT17 also diminished the downstream proteins included within Wnt/*β*-catenin pathway, such as cyclin D1, c-Myc, and MMP7. Importantly, the western blot assay also found the expression of mesenchymal marker (Snail) and cell cycle-related proteins, counting CDK2, CDK4, and Cyclin E, was diminished in the sh-KRT17 group compared to the sh-NC group. Taken together, our discoveries uncovered that KRT17 actuated LSCC progression, at least in part by means of enactment of AKT/mTOR and Wnt/*β*-catenin axes.

## 4. Discussion

Patients diagnosed with LSCC can be treated by surgery, chemotherapy, or radiotherapy, but the overall therapeutic impact is still not palatable, the long-term survival rate is low, and the prognosis is destitute. The occurrence and development of LSCC is considered to be the result of the imbalance between oncogenes and tumor suppressor genes. In this manner, effectively seeking gene-level therapeutic targets and potential mechanisms has become the focus of current research. In this study, we examined the relevance of KRT17 to LSCC. Our research recommended that KRT17 regulates growth, migration, and invasion in LSCC through the AKT/mTOR and Wnt/*β*-catenin axes. These discoveries uncover that KRT17 plays a part in the advancement of LSCC and is probably a prospective therapy candidate.

Studies have appeared that KRT17 can directly participate in the regulation of an assortment of tumors. For example, KRT17 impacts the pathogenesis of cervical cancer by promoting the nuclear transport and degradation of protein P27 (Kip1) [25]. Mihaela et al. detailed that knockdown of KRT17 diminished cell motility and invasion capacity and retards tumor growth through p38, AKT/mTOR and ERK/JNK signaling in gastric cancer [12]. Yan et al. revealed that KRT17 regulates osteosarcoma cell proliferation, glycolysis, and tumor development through AKT/mTOR/HIF1*α* pathway [21]. KRT17 was found to play a key role in advancing cervical cancer development and paclitaxel-induced mediate resistance [26]. Moreover, KRT17 advances carcinogenesis of skin cancer [27], non-small-cell lung cancer [22], renal cell carcinoma [28], pancreatic cancer [29], and hepatocellular carcinoma [16]. Li et al. detailed that KRT17 was included in areca-induced oral cancer [18], and Khanom et al. showed that KRT17 promoted oral cancer tumor growth [17]. These discoveries imply an oncogenic role of KRT17, but its exact function in LSCC remains to be clarified.

We obtained high expression of KRT17 mRNA in LSCC by comparing 42 pairs of LSCC tissues with normal adjacent tissues, which is consistent with the present study [19]. KRT17 has been proven to be a prognostic marker for a multitude of cancers, including lung cancer [22], colon cancer [30], cervical cancer [31], bladder cancer [32], etc. The ability to predict outcomes and to identify key players in biological mechanisms that lead to poor outcomes are two important objectives in cancer research [15]. In LSCC, we ascertained that expression of KRT17 in a panel of LSCC samples (42 cases) was positively correlated with the differentiation, T-typing, lymph node metastasis, and clinical stage. Noticeably, overall survival analysis indicated that patients with high KRT17 expression levels exhibited a remarkable shorter survival duration than those displaying low KRT17 expression levels, recommending its potential as a prognostic marker of LSCC. We further affirmed the part of KRT17 in LSCC cells. Depletion of KRT17 hindered cell proliferative, migratory, invasive abilities and contributed to cell cycle arrest. Additionally, in vivo xenograft tumor studies inferred that knockdown of KRT17 clearly repressed tumorigenesis, i.e., lower tumor volume and weight, and Ki-67 level. Hence, KRT17 plays an imperative part in advancing LSCC proliferation and metastasis.

Activation of AKT/mTOR and Wnt/*β*-catenin axes in LSCC has been extensively studied [33–36]. Past studies have suggested that KRT17 mediates cancer progression through the AKT/mTOR or Wnt/*β*-catenin axis [21, 22]. In this manner, we focused on KRT17 in LSCC mechanisms through the above-mentioned signals. Western blot revealed that downregulation of KRT17 essentially diminished the expression of p-AKT, p-mTOR, and *β*-catenin, which affirmed the inactivation status of the AKT/mTOR and Wnt/*β*-catenin axes. Consistently, the downstream proteins of the Wnt/*β*-catenin axis and the cell cycle-related proteins were also diminished after the exhaustion of KRT17.

## 5. Conclusion

In conclusion, our study demonstrates that the knockdown of KRT17 restrains proliferation, migration, and invasion partly through AKT/mTOR and Wnt/*β*-catenin axes, proposing KRT17 as a novel biological target in LSCC and giving unused considerations for the alter of treatment of LSCC.

## Figures and Tables

**Table 1:** Primer sequences.

Gene	Forward primer	Reverse primer
KRT17	5′-GATCCGTGACTGGTACCAG-3′	5′-TGTGAGGATCTTGTTCTGCA -3′
GAPDH	5′-TCAAGATCATCAGCAATGCC-3′	5′-CGATACCAAAGTTGTCATGGA-3′

Protein-protein interaction (PPI) network construction.

**Table 2:** Correlation between KRT17 and clinicopathological features of LSCC patients (*n* = 42).

Characteristic	No. (*n* = 42)	KRT17 expression level		*P* value
		Low (*n* = 23)	High (*n* = 19)	
Gender				0.845
Female	17	9	8	
Male	25	14	11	

Primary location				0.248
Supraglottic	27	13	14	
Glottic	15	10	5	

T classification				0.001^*∗∗∗*^
T1 + T2	20	17	3	
T3 + T4	22	6	16	

Differentiation				0.002^*∗∗*^
High	20	6	14	
Moderate + poor	22	17	5	

Lymph node metastasis				0.016^*∗*^
Yes	18	6	12	
No	24	17	7	

Clinical stage				0.03^*∗*^
I + II	21	15	6	
III + IV	21	8	13	

^*∗*^A significant difference compared with the KRT17 low expression group. ^*∗*^*P* < 0.05, ^*∗∗*^*P* < 0.01, and ^*∗∗∗*^*P* < 0.001.

## Data Availability

All data generated or analyzed during this study are included in this article.
